# GeneXplorer: an interactive web application for microarray data visualization and analysis

**DOI:** 10.1186/1471-2105-5-141

**Published:** 2004-10-01

**Authors:** Christian A Rees, Janos Demeter, John C Matese, David Botstein, Gavin Sherlock

**Affiliations:** 1 Dept. of Genetics, 300 Pasteur Drive, Stanford University Medical School, Stanford, CA 94305-5120, USA; 2 Dept. of Biochemistry, Stanford University School of Medicine, Stanford, CA 94305-5307, USA; 3 Lewis-Sigler Institute for Integrative Genomics Carl Icahn Laboratory, Princeton University, Princeton, NJ 08544, USA

## Abstract

**Background:**

When publishing large-scale microarray datasets, it is of great value to create supplemental websites where either the full data, or selected subsets corresponding to figures within the paper, can be browsed. We set out to create a CGI application containing many of the features of some of the existing standalone software for the visualization of clustered microarray data.

**Results:**

We present GeneXplorer, a web application for interactive microarray data visualization and analysis in a web environment. GeneXplorer allows users to browse a microarray dataset in an intuitive fashion. It provides simple access to microarray data over the Internet and uses only HTML and JavaScript to display graphic and annotation information. It provides radar and zoom views of the data, allows display of the nearest neighbors to a gene expression vector based on their Pearson correlations and provides the ability to search gene annotation fields.

**Conclusions:**

The software is released under the permissive MIT Open Source license, and the complete documentation and the entire source code are freely available for download from CPAN .

## Background

Microarray experiments produce vast amounts of data. The resulting datasets are highly complex and contain large matrices of expression measurements as well as sequence and experiment annotations that provide biological context to the data. To organize these different types of data in a way that allows intuitive exploration of the data, and provides the ability to gain important insights into relationships within a given dataset requires sophisticated visualization tools. Such visualization tools are of benefit not only to researchers analyzing and presenting or publishing their own data, but also to Model Organism Databases (MODs) for compiling and displaying microarray data for a given model organism.

There are several excellent free tools available that allow an individual user to analyze their own data. These tools are either accessible on the web, or can be downloaded and used on a desktop machine. Examples include the EPCLUST [1], GEPAS [2,3] and FGDP [4,5] web-based tools and the TMEV [6,7] desktop tool from TIGR. However, once these tools have been used, and a cluster or other group of genes has been selected, this resulting dataset needs to be made available to other people for browsing and exploration. There are a few visualization tools that allow display of such a static dataset that are available as free software tools, e.g. Michael Eisen's TreeView [8,9], JavaTreeView [10], or the more recent MapleTree [8]. All of these tools are, however, desktop tools that themselves have to be downloaded and work on locally stored datasets. The impetus for the development of GeneXplorer was the desire to provide access to datasets via the Internet, without the requirement to download and install additional software. We developed GeneXplorer for use in web supplements of microarray publications whose raw data are housed within the Stanford Microarray Database (SMD) [11,12] and for use as a tool to allow SMD users to browse their own data within SMD before publication. Using GeneXplorer, hierarchically clustered gene expression data can be interactively viewed using a web browser on any computer platform. GeneXplorer uses the widely accepted CDT file format [13] produced by several freely available clustering programs (e.g. [9,14]), which between them have been downloaded several thousand times. Thus GeneXplorer should be widely usable my SMD and non-SMD users alike.

## Implementation

**Figure 1:**
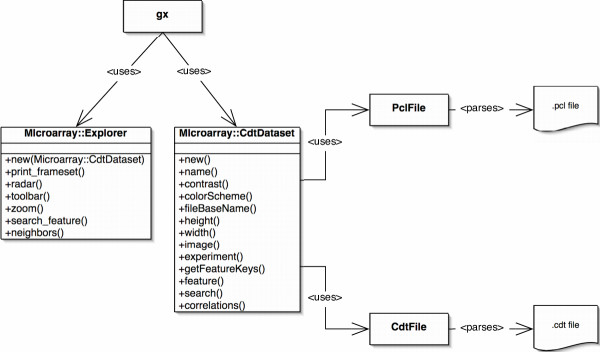
**Simplified UML diagram showing the main components of GeneXplorer. **The Controller, gx, creates a Microarray::CdtDataset (the Model), which it then uses in construction of a Microarray::GeneXplorer object (the View), which render the Model through a web browser. Microarray::CdtDataset uses PclFile and CdtFile objects, which in turn provide abstraction layers to either a .pcl or a .cdt file, respectively.

The application was written using object oriented Perl following the Model-View-Controller (MVC) design paradigm [15]. GeneXplorer consists of two classes, the data model class Microarray::CdtDataset (M), and the presentation logic class Microarray::Explorer (V). The controller, named gx, is a Perl CGI script that dispatches CGI requests to the viewer. The MVC paradigm was used because it dissociates how data are represented internally (the Model) from how they are displayed (the View), from how they are interacted with (the Controller) (see Figure 1.). The goal of such a separation is that by keeping consistent APIs for the components to interact with each other, each component may be modified extensively internally, with little or no effect on the other parts of the application, thus making code maintenance easier. The Microarray::CdtDataset class provides an application programming interface (API) that allows details of a particular expression cluster to be queried. In turn, instances of the Microarray::Explorer class use this API to retrieve and then display information about the dataset. The controller is a relatively simple CGI Perl script that is responsible for capturing CGI parameters and using them to first create a dataset Microarray::CdtDataset object, which is subsequently used in the instantiation of a Microarray::Explorer object. The controller then invokes the appropriate Microarray::Explorer methods, depending on where, and in which frame the user clicked.

The Microarray::CdtDataset has two essential functions: during dataset creation (see below) it decomposes the data file into its constituent data parts and creates the files needed during data viewing (see below). During data viewing it provides the API for the viewer, and allows searching and retrieval of the data. Under the current model the dataset object itself is immutable. Microarray::CdtDataset was implemented as a client of the Microarray::DataMatrix module, which provides an API for accessing matrices of expression data. In the design of the classes certain compromises had to be made to accommodate the stateless client server environment in which the program operates. Specifically, to allow rapid responses, pre-generated images and correlation data are cached in a compact format on the web-server.

There are two stages required to publish a microarray dataset on the web using GeneXplorer. The first stage (executed only once per dataset) involves creation of all the necessary files for GeneXplorer to use. The second stage uses these files to produce the display using the GeneXplorer web front-end.

### Dataset creation

**Figure 2:**
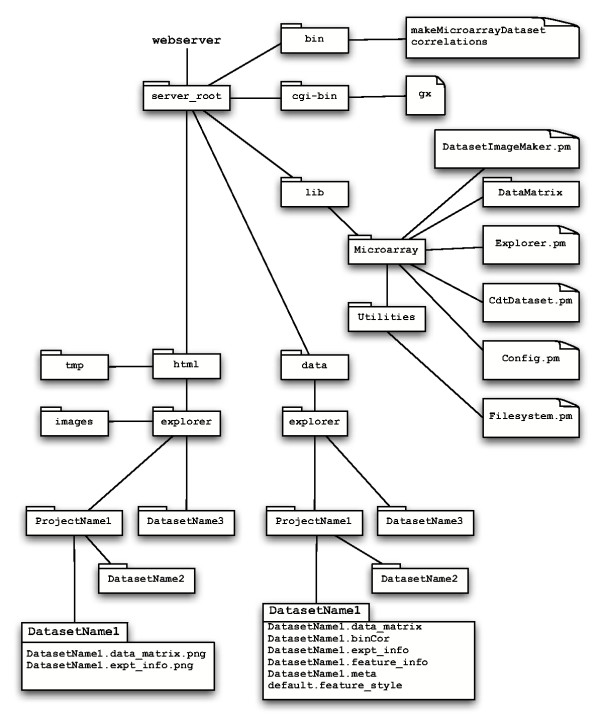
**The file system diagram used by GeneXplorer. **Programs within the GeneXplorer distribution assume a certain directory structure during dataset creation, and subsequently during display by the gx application. For dataset creation /html /explorer/ and /data/explorer directories are assumed and dataset specific directories are created under each one of them. For data display web accessible directories are required for reading general images (html/explorer/images/) and images used for a particular dataset (html/explorer/datasets), as well as for creating temporary images (html/tmp). A cgi-bin directory where the gx application itself will exist, and a directory in which the dataset files can be stored and read by the cgi application are also assumed.

Dataset creation requires a file in the Clustered Data Table (CDT) format: a simple tab delimited text file format (see [13] for file format details). This format was introduced with the 'Cluster' and 'TreeView' applications [8] and is widely used for microarray data. A perl script (makeMicroarrayDataset.pl) uses Microarray::CdtDataset to create the various required data files. Correlations between expression-vectors within the dataset are calculated for each pair-wise combination of vectors using the C program 'correlations'. Correlations for each vector above the default cutoff value of 0.5 (which is configurable) are saved in a binary format that facilitates rapid searching. Depending on the version of the Perl GD module [16] installed on the system, either png or gif formatted images representing the cluster will be created. These images include both a 2-color image representation of the data matrix and an image representation of the experiment names. The program that creates these files is configurable, such that these images can be created using either a red/green or a yellow/blue color scheme, and in addition, the contrast of the images can be customized and set in steps of log(2) scale. The name and path of a dataset can be defined hierarchically within the file system (see Figure 2) allowing the creation of many datasets within the same project.

### Dataset viewing

**Figure 3:**
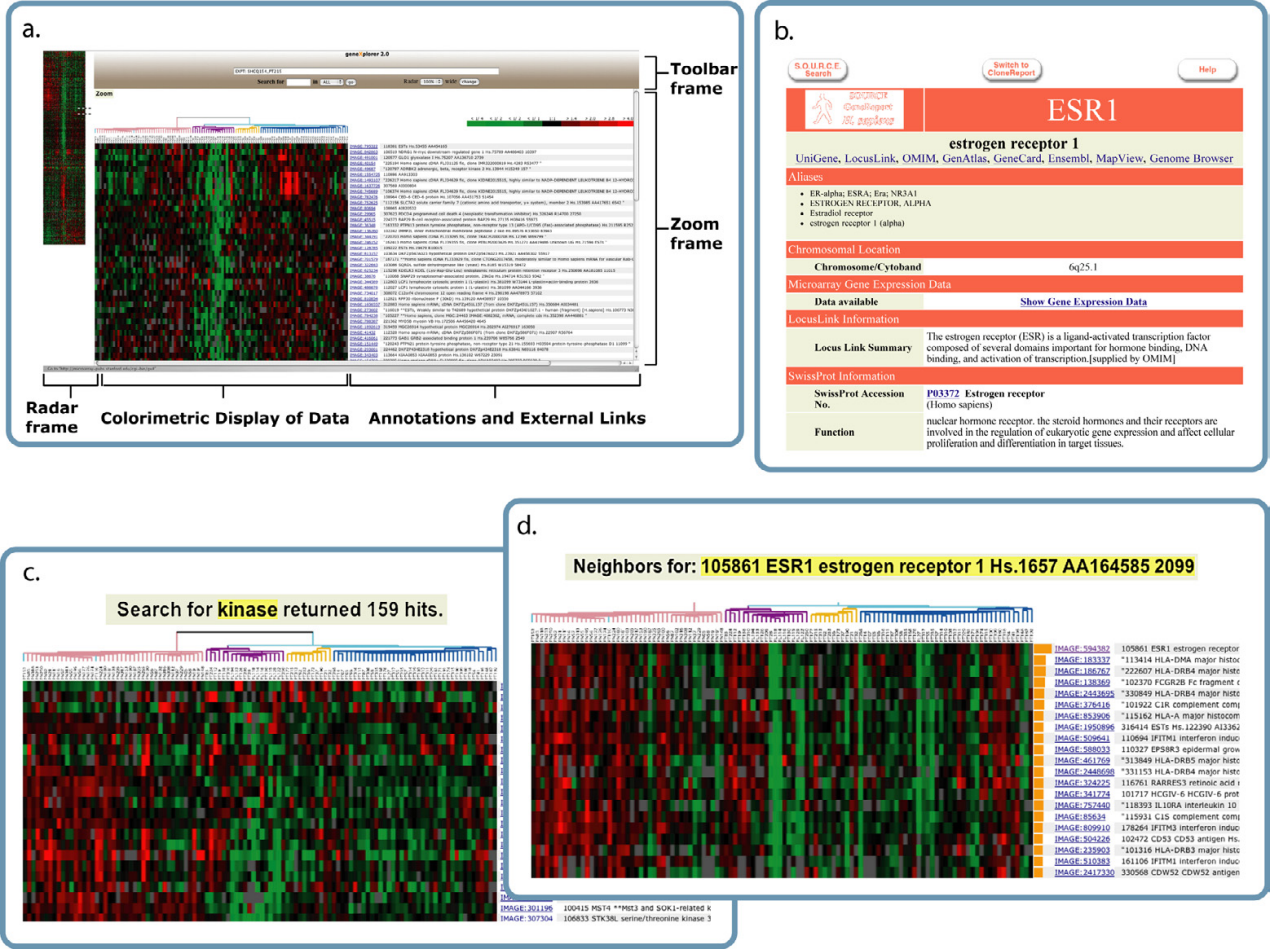
**Visualization of a dataset by GeneXplorer. **a.) Overview of GeneXplorer display, explaining the various parts of the window. b.) In the zoom frame, the gene annotations table may contain an unlimited number of graphics and hyperlinks regarding the genes. These are configurable via a server-side stylesheet to accommodate different organism annotation. Clicking on these hyperlinks can take the user to e.g. SOURCE – on online collection of information about mammalian genes/clones. c.) SEARCH result display in zoom window. The search tool in the tool frame allows searching the annotations by any or all of the fields by using either words or wildcard searches. The result is displayed in the zoom window. d.) GeneXplorer's gene correlation display. The genes with expression patterns similar to the gene of interest are presented in an ordered list. The length of the orange bar to the right of the expression data indicates the magnitude of the correlation value.

GeneXplorer is a Perl application that produces a set of html frames that can be used for viewing the expression data (Figure 3.). The three frames that it produces are: 1) A radar frame. This frame displays an image map of the data matrix and gives an overall view of the clustered data. The rows correspond to the features or genes (also referred to as reporters), and the columns correspond to the experiments within the dataset. When the image is clicked the next 100 expression patterns starting at the position of the click are displayed in the zoom frame. The position of a bracket on the right side of the radar window indicates the section of the whole radar image that is displayed in the zoom frame. 2) A toolbar frame. Actions in the toolbar may affect either the radar or the zoom frame. There is a tool to set the scaling of the radar image, while the search box allows searching of gene annotations and the expression patterns of the resulting hits are displayed in the zoom frame. In addition the toolbar frame also contains a JavaScript enabled text box that gives feedback depending on the user's mouse position, to provide additional information about the genes and experiments within the cluster. 3) A zoom frame. This frame displays a zoomed view of selected expression patterns, such that the user can see both the individual patterns and the associated annotations. The source of the selected patterns can be either a section of the radar image, the result of a search the user performed in the toolbar, or the result of a nearest neighbor search initiated in the zoom frame itself. The expression profiles themselves in the zoom frame are clickable and the resulting search will display the expression pattern for the most similarly expressed genes to the gene that was clicked on, and provide visual feedback as to the level of similarity in their expression profiles. In addition, when the user moves the mouse over parts of the zoom window, additional information is directed back to the textbox in the toolbar. The experiment name, correlation value (Figure 3d) and gene annotation is displayed when the mouse is over the experiment image map, the correlation bar, and the expression pattern, respectively.

### Full text searching

The search box in the toolbar enables a string search of either all, or specific gene annotation fields. The string may contain more than one term, where each term in the search string should be at least 2 characters long. Spaces between the terms are interpreted as term separators and the terms are combined using the logical 'AND' operator. Wildcard searches are allowed using the '*' character, such that at least one character should precede the wildcard character. The hits resulting from the search are displayed in the zoom frame, as expression patterns. The number of hits displayed in the zoom window is limited to 200 hits.

### Display configuration

GeneXplorer allows configurable linking out of the gene annotations to external databases. The number of these links per a gene is not limited, making it easy to be able to look at the information for a gene in several different databases. A configuration file in the dataset directory is used to control where the various gene identifiers are linked. Templates are available for various organisms, and the existing files can be edited manually if a link to a new database is desired. Because of the current limitations of the input cdt file format, setting up the external database links might require manual editing at the time of dataset creation. This is fully described in the README document that is part of the distribution. The external database annotations are not currently updatable in any automated fashion; this will be addressed as part of our plans to make GeneXplorer able to read MAGE-ML (see future plans) that would allow us to do the updates via web services.

### Installation and use

The GeneXplorer package is provided as a typical Perl distribution on the Comprehensive Perl Archive Network (CPAN), and adheres to the usual installation mantra of perl modules. After unpacking the software, a user with administrative privileges merely needs to type:

perl Makefile.PL

make

make test

make install

This will install the libraries and the executable files that are needed for dataset creation by GeneXplorer into the regular system locations, unless otherwise specified during the first step above. The example in Figure 2 shows the file structure if the library and bin directories under the web server's root had been specified for installation of the libraries and executables respectively. To actually use the gx script, it must be copied into a cgi-bin directory, and the various html files must be copied to the appropriate location under the web server's root (see Figure 2).

## Results and discussion

In addition to its use within SMD, GeneXplorer has been used by many publications to provide access to microarray datasets through their web supplements, that can be accessed through SMD's publication page [17], and was used as the basis for visualization of fuzzy k-means cluster data [18]. We demonstrate on an example dataset how GeneXplorer works [19,20]. Figure 3a shows a display of this dataset in the browser window. The whole dataset is displayed in the radar frame, and the zoom window shows the section of this image that was selected, with the gene annotations at a readable size. Clicking on any of the hyperlinks in the zoom frame brings up a new window displaying the biological information for the selected gene that is found in SOURCE (Figure 3b.) [21]. Searching the dataset for all the genes whose name field contains the keyword 'kinase' results in the zoom window shown in Figure 3c. This type of search allows comparison of the expression patterns of a subset of the genes based on some functional category – e.g. GO process-terms, if the annotation fields contain these terms. Clicking on one of the expression profiles (the one belonging to 'Estrogen Receptor 1', in this case) leads to the display in Figure 3d. In the zoom frame it shows the expression profile of the selected gene as the top row, and all the other expression profiles below with Pearson correlation above 0.5. The length of the small orange bar on the right side of the expression profiles gives a graphical representation of these correlation values, while the actual value is displayed in the info box in the toolbar when the mouse is over the orange bar.

### Future developments

We are planning to further develop GeneXplorer to enable it to handle other data formats. Specifically, we would like it to be able to accept data files in MAGE-ML format [22], which is becoming a standard file format for communicating gene expression data. In addition, we would like it to be able to display tree views of the clustered data and allow zooming on specific nodes of the cluster.

## Conclusions

We have developed a web-application, GeneXplorer, which allows the visualization of microarray datasets over the Internet using only a web browser. This application has been extremely useful in our experience, where it serves both SMD users during analysis of their data and the public while browsing published datasets.

## Availability and requirements

GeneXplorer is available at [23] under the MIT Open Source license. It should work on any UNIX-type system capable of running Perl and a Web server, though we ourselves have deployed it on Sun Solaris. Additional information on installation and usage is provided in the installation instructions and documentation that is part of the distribution.

## List of abbreviations used

SMD: Stanford Microarray Database.

## Authors' contributions

CAR designed and wrote the initial version of the GeneXplorer. This was extensively re-factored and modularized by JCM (library modules for dataset) and JD (for explorer). DB was involved in the guidance of the early stages of this project. GS wrote the correlations software and the DataMatrix classes, and guided the development of this project. All authors read and approved the final version of the manuscript.
